# Ginkgo biloba leaf extract improves the cognitive abilities of rats with *D*-galactose induced dementia

**DOI:** 10.7555/JBR.27.20120047

**Published:** 2012-12-20

**Authors:** Nuan Wang, Xianming Chen, Deqin Geng, Hongli Huang, Hao Zhou

**Affiliations:** a Department of Neurology, the First People's Hospital, Xuzhou, Jiangsu 221002, China;; b Department of Neurology, the Affiliated Hospital of Xuzhou Medical College, Xuzhou, Jiangsu 221002, China;; c Department of Neurology, the 73087 Military Hospital, Xuzhou, Jiangsu 221002, China.

**Keywords:** dementia model, *D*-galactose, Ginkgo biloba extract, apoptosis, protein kinase B (PKB), cognitive ability

## Abstract

Standardized Ginkgo biloba leaf extract has been used in clinical trials for its beneficial effects on brain functions, particularly in dementia. Substantial experimental evidences indicated that Ginkgo biloba leaf extract (EGB) protected neuronal cells from a variety of insults. We investigated the effect of EGB on cognitive ability and protein kinase B (PKB) activity in hippocampal neuronal cells of dementia model rats. Rats received an intraperitoneal injection of *D*-galactose to induce dementia. Forty-eight Spraque-Dawley rats were randomly divided into six groups, including the control group, *D*-galactose group (Gal), low-dose EGB group (EGB-L), mid-dose EGB group (EGB-M), high-dose EGB group (EGB-H) and treatment group. The EGB-L, EGB-M and EGB-H groups were administered with EGB and *D*-galactose simultaneously. Y-maze, cresyl violet staining, TUNEL assays and immunohistochemistry staining were performed to detect learning and memory abilities, morphological changes in the hippocampus, neuronal apoptosis and the expressing level of phospho-PKB, respectively. Rats in the Gal group showed decreased abilities of learning and memory, and hippocampal pyramidal cell layer was damaged, while EGB administration improved learning and memory abilities. The Gal group exhibited many stained, condensed nuclei and micronuclei, either isolated or within the cytoplasm of cells (39.5±1.4). Apoptotic cells decreased in the groups of EGB-L (35.9±0.9), EGB-M (16.8±1.0) and EGB-H (10.1±0.8), and there were statistical significances compared with the Gal group. Immunoreactivity of phospho-PKB was localized diffusely throughout the cytosol of cells in all groups, while the immunoreactivity of the Gal group was weak. EGB significantly attenuated learning and memory impairment in a dose-dependent manner, while it could decrease the nmber of TUNEL-positive cells, and increase the activity of PKB. Our results demonstrated that EGB attenuated memory impairment and cell apoptosis in galactose-induced dementia model rats by activating PKB.

## INTRODUCTION

Dementia is characterized by progressive loss of memory and thinking abilities, resulting in a gradual deterioration in social and occupational functioning. Chronic systemic exposure of rats to *D*-galactose causes the acceleration of senescence and *D*-galactose-induced dementia has been used as an aging model[1]–[3]. The aging model shows neurological impairment, decreased activity of anti-oxidant enzymes and poor immune responses[4]. Ginkgo biloba leaf extract has been marketed as a therapeutic agent to counteract a variety of neurological disorders[5]. Pharmacological studies have shown that Ginkgo biloba leaf extract increased cerebral blood flow in a focal ischemia model[6] and attenuated 1-methyl-4-phenyl-1, 2, 3, 6 tetrahydropyridine induced nigrostriatal dopaminergic neurotoxicity in C57 mice[7].

Recently, placebo-controlled, double-blind and randomized trials have demonstrated that standardized Ginkgo biloba leaf extract 761 is effective in mild-to-moderate dementia patients[8],[9]. However, the neuroprotective mechanisms of Ginkgo biloba leaf extract remain not completely clear[10]. Apoptosis is implicated in the pathology of dementia[11],[12]. Protein kinase B (PKB) plays a critical role in the promotion of cell survival[13] and inhibition of the activity of caspase family members, which result in caspase-dependent cell death[14]–[16]. These findings suggested that PKB could be potential pharmacological targets in strategies aimed at slowing progression of neuronal loss in dementia. In the present study, we investigated the mechanisms of neurodegeneration in galactose-induced dementia model rats by studying cognitive function, hippocampal neuronal apoptosis and PKB activity. Further, we observed the protective effects of Ginkgo biloba leaf extract on *D*-galactose-induced dementia and explored the protective mechanisms of Ginkgo biloba leaf extract.

## MATERIALS AND METHODS

### Animals

Sprague-Dawley (SD) rats, weighing 280-350 g, were obtained from the Experimental Animal Center of Xuzhou Medical College (Xuzhou, Jiangsu, China). All experiments were performed in accordance with the protocols approved by the University Committee on Animal Care of Xuzhou Medical College.

### Reagents

Anti-phospho-PKB and HRP-conjugated secondary antibodies were obtained from Santa Cruz Biotechnology (Santa Cruz, CA, USA). Immunohistochemistry assay kit, TUNEL assay kit and Ginkgo biloba leaf extract were obtained from Booster Co. (Wuhan, Hubei, China). Ginkgo biloba leaf extract is a complex mixture containing 24% flavonoids (e.g., kaempferol, quercetin and isorhamnetin derivatives), 6% terpenes (e.g., ginkgolides A, B, C, J and bilobalide) and other various constituents.

### Animal groups

Forty-eight rats were randomly divided into six groups after training by Y-electric maze. Rats in the control group (*n* = 8) were injected with an equal volume of saline. Rats in the galactose group (Gal *n* = 8) were treated with *D*-galactose (100 mg/kg, peritoneal injection each day). Rats in the low-dose Ginkgo biloba leaf extract group (0.875 mg/kg, EGB-L *n* = 8), the middle-dose Ginkgo biloba leaf extract group (1.75 mg/kg, EGB-M *n* = 8) and the high-dose Ginkgo biloba leaf extract group (3.5 mg/kg, EGB-H *n* = 8) were administered with Ginkgo biloba leaf extract and *D*-galactose (100 mg/kg) each day by peritoneal injection for 8 weeks. Rats in the treatment group were administered with *D*-galactose (100 mg/kg) for 8 weeks and then treated with Ginkgo biloba leaf extract (1.75 mg/kg) for 2 weeks.

### Behavioral test

Learning and memory abilities were assessed by Y-maze. The Y-maze consists of three equal arms (40 ×15×15 cm) with a stainless-steel grid floor. After a 1-min habituation period in maze, the rats were given a foot shock (50-70 mV) in the start arm and then escaped to other arms. If rats ran to the right arm (correct), they would receive no foot shock. If rats entered into the left arm (error), they would receive further foot shock and stop running when they entered the right arm. The rats were not given trials until they could escape to the right arm nine times for ten continuous trials (9/10), and trial numbers were recorded as learning ability. Twenty-four hours after training session, retention of Y-maze task was tested using the same procedure and correct numbers per ten trials were recorded as memory ability. Tests were performed every 2 weeks and carried out in similar environmental conditions.

### Cresyl violet staining, TUNEL and Immunohistochemistry

Rats (10 weeks) were anesthetized with chloral hydrate and underwent transcardial perfusion with cold saline followed by 4% paraformaldehyde in 0.1 mol/L phosphate buffered saline (PBS). Brains were removed and post-fixed in 4% paraformaldehyde in 0.1 mol/L PBS overnight for paraffin embedding. Coronal brain sections (5 um thick) were cut by a microtome (Leica, RM2155; Nussloch, Germany). Sections were deparaffinized in xylene, rehydrated in ethanol and distilled water. For Cresyl violet staining, sections were stained with 0.1% cresyl violet for 30 minutes for assessment of neuronal damage in CA1 region of the hippocampus under microscopy. For immunohistochemistry and TUNEL, The sections were stained by introduction of reagent kit. TUNEL-positive cells were counted in five non-overlapping visual fields for each section under light microscopy.

**Table 1:** Changes of learning ability in different groups of rats (trial numbers)

Groups	Before treatment	After teatment			
		2 weeks	4 weeks	6 weeks	8 weeks
Control	46.7±8.2	45.0±5.5	48.3±4.1	46.7±5.2	50.0±6.3
Gal	50.0±8.9	55.0±5.5	60.0±8.9	70.0±8.9**	76.7±5.2**
EGB-L	48.3±7.5	53.3±8.2	55.0±5.5	68.3±7.5**	73.3±8.2**
EGB-M	45.0±8.4	48.3±7.5	53.3±5.2	53.3±5.2^##^	56.7±8.2^##^
EGB-H	45.0±5.5	50.0±6.3	51.2±7.5	50.0±6.3^##^	51.7±4.1^##^

Gal: rats in the galactose group were treated with *D*-galactose (100 mg/kg, peritoneal injection each day). EGB-L: rats in the low-dose Ginkgo biloba leaf extract group received 0.875 mg/kg Ginkgo biloba leaf extract. EGB-M: rats in the middle-dose Ginkgo biloba leaf extract group received 1.75 mg/kg Ginkgo biloba leaf extract. EGB-H, rats in the high-dose Ginkgo biloba leaf extract group received 3.5 mg/kg biloba leaf extract. *P < 0.05, **P < 0.01 vs Control group; ^#^*P* < 0.05, ^##^*P* < 0.01 vs Gal group.

### Statistical analysis

All data were expressed as mean±SEM, statistics were performed with the statistical SPSS software package (version 11.0, SPSS Inc, Chicago, IL, USA). Differences among groups were compared by one-way analysis of variance (ANOVA). The two groups were compared using t test. Differences were deemed statistically significant if *P* < 0.05.

## RESULTS

### Effects of Ginkgo biloba leaf extract on learning and memory abilities of rats with *D*-galactose-induced dementia

To determine whether Ginkgo biloba leaf extract affected learning and memory abilities of rats with *D*-galactose-induced dementia, we measured trial numbers during the training (learning) and correct numbers per ten trials (memory numbers) during the retention session (memory) by Y-maze. Our data (***Table 1*** and ***Table 2***) showed that rats in the Gal group showed increased trial numbers and decreased memory numbers compared with the control group after *D*-galactose administration for 6 weeks (*P* < 0.01), while Ginkgo biloba leaf extract significantly attenuated learning and memory impairment in a dose-dependent manner, especially in the EGB-M and the EGB-H groups. Six weeks after treatment, trial numbers in the Gal group were 70.0±8.9 while in the EGB-M group were 53.3±5.2 and in the EGB-H group were 50.0±6.3. Memory numbers in the Gal group were 5.0±0.9 while in the EGB-M group were 6.7±0.5 and in the EGB-H group were 6.8±0.4, with statistical significant difference in all groups. In the therapy group, learning and memory abilities were also improved after Ginkgo biloba leaf extract treatment for 2 weeks. Trial numbers before treatment were 76.7±5.2 and were 65.0±5.5, 2 weeks after treatment and memory numbers before treatment were 3.7±0.8 and were 5.7±0.52 weeks after treatment. The differences all had statistical significance.

**Table 2:** Changes of memory ability in different groups of rats (memory numbers)

Groups	Before treatment	After teatment			
		2 weeks	4 weeks	6 weeks	8 weeks
Control	8.0±0.9	7.3±0.5	7.5±0.5	7.7±0.8	7.7±0.5
Gal	7.8±1.0	7.0±0.9	5.8±0.8	5.0±0.9**	3.7±0.8**
EGB-L	7.7±0.5	6.8±0.8	6.0±0.9	5.2±1.0**	4.2±1.0**
EGB-M	8.2±0.8	7.2±0.4	6.3±0.8	6.7±0.5^#^	6.5±0.5^##^
EGB-H	8.3±0.8	7.2±0.8	6.5±0.8	6.8±0.4^##^	6.7±0.5^##^

Gal: rats in the galactose group were treated with *D*-galactose (100 mg/kg, peritoneal injection each day).EGB-L: rats in the low-dose. Ginkgo biloba leaf extract group received 0.875 mg/kg Ginkgo biloba leaf extract. EGB-M: rats in the middle-dose Ginkgo biloba leaf extract group re-ceived 1.75 mg/kg Ginkgo biloba leaf extract. EGB-H, rats in the high-dose Ginkgo biloba leaf extract group received 3.5 mg/kg biloba leaf extract. *P < 0.05, **P < 0.01 vs Control group; ^#^*P* < 0.05, ^##^*P* < 0.01 vs Gal group.

### Effects of EGB on morphology of CA1 region of the hippocampus of rats with dementia

**Fig. 1:**
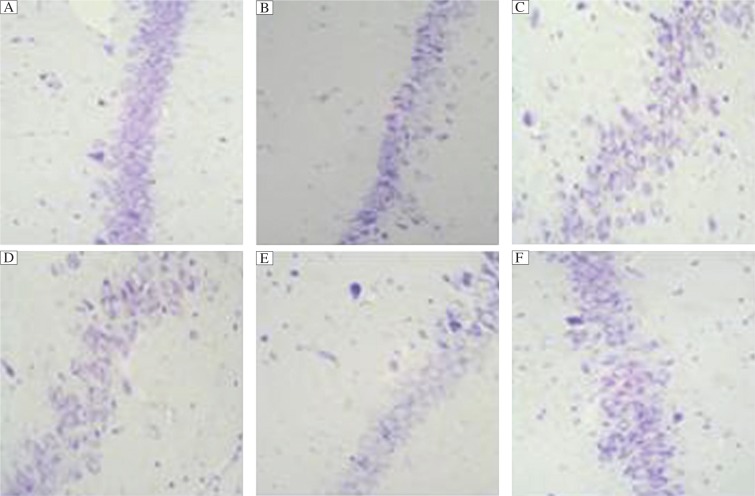
Representative cresyl violet-stained coronal sections of the hippocampus from rats (×400). Dementia in rats was induced with *D*-galactose. The rats were then treated with Ginkgo biloba leaf extract as detailed in Materials and Methods. A: Control group; B: *D*-galactose (Gal) group; C: EGB-L group; D: EGB-M group; E: EGB-H group; F: Therapy group. Normal arrangement and number of cells are seen in the hippocampus of the control group. The sections of the Gal group and treatment group show many cells with pyknotic nuclei and cytoplasm and they are deranged, especially for the Gal group. The sections from the groups of EGB-L and EGB-M demonstrate fewer cells with pyknotic nuclei and cytoplasm. Cells from the EGB-H group are identical to control, except for several deranged cells.

Brain injury in dementia rats was evaluated by cresyl violet staining. Representative histological preparations showed that the morphology of the hippocampus of six groups were essentially identical, except for the number and arrangement of cells. High magnification (400×light microscopy) of cresyl violet staining demonstrated the most cells with pyknotic nuclei and chromatin clumping for the Gal and therapy groups, especially for the Gal group, while the groups of EGB-L, EGB-M and EGB-H showed fewer cells with pyknotic nuclei and chromatin clumping dose-dependently. Hippocampal cells of the conrol group were normal. (***Fig. 1***)

### Effects of Ginkgo biloba leaf extract on neuronal apoptosis in CA1 region of the hippocampus of rats with dementia

**Fig. 2:**
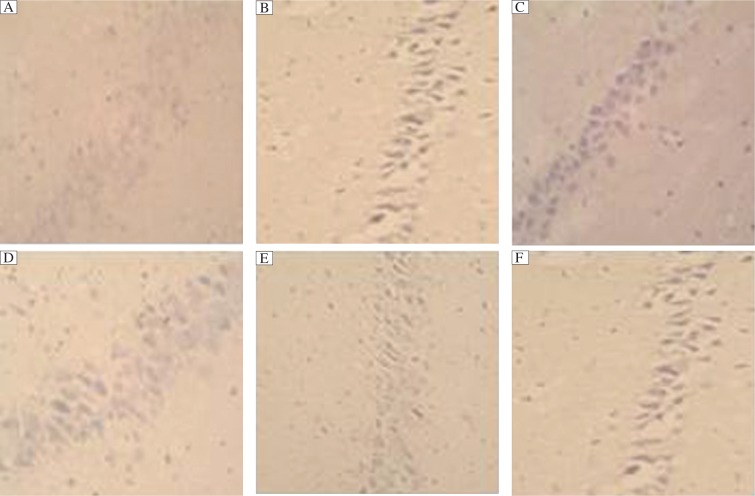
Effects of Ginkgo biloba leaf extract on apoptosis of cells in the hippocampus of rats with *D*-galactose induced dementia (×400). A: Control group; B: *D*-galactose (Gal) group; C: EGB-L group; D: EGB-M group; E: EGB-H group; F: Therapy group. Representative TUNEL-stained coronal sections (5 fields per section, *n*=8) of the hippocampus from rats. Apoptotic cells have classic condensation and fragmentation of their nuclei.

TUNEL staining assays demonstrated that the Gal group exhibited many stained, condensed nuclei and micronuclei, either isolated or within the cytoplasm of cells (39.5±1.4 per field of 400×light microscopy). There was significant difference compared with the control group (*P* < 0.01). As shown in ***Fig. 2***, apoptotic cells had classic condensation and fragmentation of their nuclei. Lack of TdT appeared blank. Apoptotic cells decreased in the groups of EGB-L (35.9±0.9), EGB-M (16.8±1.0) and EGB-H (10.1±0.8), and there were statistical significances compared with the Gal group while the percentage of apoptotic cells in the treatment group (36.2±0.9) did not differ from the Gal group. The data are shown in ***Table 3***.

**Table 3:** TUNEL-positive cells in different groups

Group	TUNEL-positive cells
Control	0.9±0.3
Gal	39.5±1.4**
EGB-L	35.9±0.9**
EGB-M	16.8±1.0**^##^
EGB-H	10.1±0.8**^##^
Therapy	36.2±0.9**

*P < 0.05,**P < 0.01 vs Control; ^#^*P* < 0.05, ^##^*P* < 0.01 vs Gal.

### Effects of Ginkgo biloba leaf extract on PKB activity in rats with *D*-galactose induced dementia

**Fig. 3:**
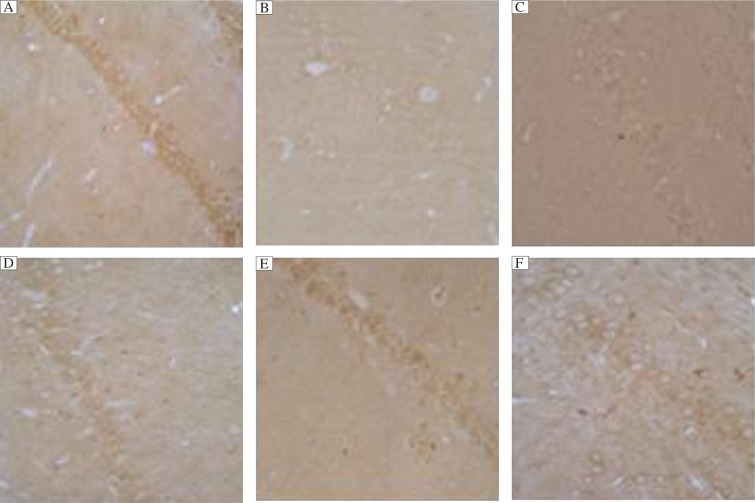
Immunohistochemical analysis of phospho-AKt (ser473) in hippocampal tissues (×400). A: Control group; B: *D*-galactose (Gal) group; C: EGB-L group; D: EGB-M group; E: EGB-H group; F: Therapy group. Representative sections from six groups showed that phospho-AKt (ser473) was localized in the cytosol of cells. The results are confirmed by immunostaining with at least four sections in every group. Immunoreactive intensity was the most intense in the control group and the weakest in the Gal group, while that of the EGB groups was more intense than the Gal group in a dose dependent manner.

To determine whether PKB activity was perturbed in the neurons of dementia rat brain and whether Ginkgo biloba leaf extract treatment for dementia rats could activate PKB activity, we investigated phospho-PKB level in the hippocampus. Ginkgo biloba leaf extract increased PKB in a dose-dependent manner. Immunoreactivity of phospho-PKB was localized diffusely throughout the cytosol of cells in the groups of Con, EGB-L, EGB-M, EGB-H and therapy, while the immunoreactivity of the Gal group was weak. (***Fig. 3***)

## DISCUSSION

*D*-galactose overload model has been used as a premature aging model[17]. Chronic systemic exposure of D-galactose to rats induced a spatial memory deficit, an increase in karyopyknosis, apoptosis and caspase-3 protein levels in hippocampal neurons, a decrease in the number of new neurons in the subgranular zone in the dentate gyrus, reduced migration of neural progenitor cells and an increase in death of newly formed neurons in the granular cell layer[1]. *D*-galactose exposure also induced an increase in oxidative stress, including an increase in malondialdehyde and tau-2 positive neurons, a decrease in total anti-oxidative capabilities, total superoxide dismutase, NT-3 positive neurons and glutathione peroxidase activities[1]. These findings suggest that chronic *D*-galactose exposure induces neurodegeneration by enhancing caspase-mediated apoptosis and inhibiting neurogenesis and neuron migration, as well as increasing oxidative damage. In addition, *D*-galactose-induced toxicity in rats is a useful model for studying the mechanisms of neurodegeneration and neuroprotective drugs and agents. Therefore, we used rats in a dementia model which was induced by intraperitoneal injection of *D*-galactose and investigated the mechanisms involved in the neuroprotective effects of Ginkgo biloba leaf extract on rat brain.

On the basis of numerous pharmacological studies on animals and more recently on humans, it has been proposed that Ginkgo biloba leaf extract ameliorates neurodegeneration associated with aging[18]. The improvements in mental efficiency and living skills shown suggested that ginkgo may be an effective treatment in the early stages of degenerative dementia. Treatment strategies from the molecular knowledge of dementia are currently in development. This study was designed to investigate the effects of Ginkgo biloba leaf extract at the cellular and molecular levels by using well-established methods of etiology, molecular biology and biochemistry. Our results demonstrated that Ginkgo biloba leaf extract may improve learning and memory abilities of rats with dementia, block apoptosis and activate PKB. Treating with Ginkgo biloba leaf extract and *D*-galactose simultaneously activated PKB, protecting the cells from apoptotic cell death. However, treatment with Ginkgo biloba leaf extract after dementia model rats were established did not cause apparent changes in the level of TUNEL-positive cells Clinical trials showed that Ginkgo biloba leaf extract[19],[20], selegiline, or -tocopherol (vitamin E) and thiamine (vitamin B1)[21] have been reported as secondary protective agents against dementia[22]. The largest known Ginkgo biloba leaf extract trial published thus far, in which primary outcome measures included the Alzheimer's Disease Assessment Scale-cognitive subscale, the Geriatric Evaluation by Relative's Rating Instrument and the Clinical Global Impression of Change, showed the superiority of Ginkgo biloba leaf extract over the placebo. In comparison to baseline values, the placebo group worsened statistically significantly regarding all domains of assessment, whereas the group receiving Ginkgo biloba leaf extract was considered slightly improved with regard to cognitive assessment and to daily living and social behavior. Regarding the safety of Ginkgo biloba leaf extract, no differences were observed[19]. Our results supported these observations and demonstrated that Ginkgo biloba leaf extract can improve the learning and memory abilities of rats with dementia detected by Y-electric maze, indicating that Ginkgo biloba leaf extract has symptomatic efficacy on dementia. Ginkgo biloba leaf extract may improve cognitive function of dementia in a number of ways. Suggested mechanisms include improving cerebral blood flow and increasing tolerance to hypoxia[23]. Ginkgo biloba leaf extract may also have a neuroprotective effect, stabilize membranes and possess anti-oxidant activity[24],[25]. In our study, we also investigated the molecular mechanism of dementia.

Apoptosis plays a critical role in normal development and maintenance of tissue homeostasis[26]. Apoptosis was found to be implicated in dementia in the early to mid-1990s[27]. In our study, dementia model of rats demonstrated many apoptotic neurons, and Ginkgo biloba leaf extract can decrease the number of apoptotic cells before dementia is achieved. Activation of PKB promotes cell survival by phosphorylating and thus inactivating a number of proapoptotic proteins and by activating anti-apoptotic proteins such as NF-kB and cAMP-response element-binding protein. Concerning neurodegeneration, the protective effect of PKB have been demonstrated against peptides of amyolid protein characteristic of senile plaques found in the brains of Alzheimer's disease patients[28] and against Parkinson inducing toxin 1-methyl-4-phenylpyridinium. In our study, Ginkgo biloba leaf extract exerts its neuroprotective effects mostly as an intracellular anti-apoptotic reagent by activating PKB, underlying the neuroprotective effect of Ginkgo biloba leaf extract. It confirms the relationship between PKB and neurodegenerative disease and the anti-apoptotic properties of Ginkgo biloba leaf extract. The results suggest that Ginkgo biloba leaf extract increases the activity of PKB by immunohistochemistry in the groups of EGB-L, EGB-M, EGB-H and therapy in a dose dependentl manner compared with the Gal group, while it can decrease the number of TUNEL-positive cells by TUNEL-staining. It indicates that before dementia is established, Ginkgo biloba leaf extract can increase the activity of PKB and inhibit apoptosis. However, our experiment showed that after the dementia model is achieved in the therapy group, the effect of Ginkgo biloba leaf extract on TUNEL-positive cells is not obvious, and the mechanisms are not fully understood.

In summary, the results indicated that Ginkgo biloba leaf extract could inhibit apoptosis by activating PKB and improve learning and memory abilities for dementia model rats. However, Ginkgo biloba leaf extract does not affect TUNEL-positive cells and of the hippocampus for the therapy group. Our conclusion is consistent with recent results of genome-wide monitoring of the biochemical effects of herbal remedies in mice, in which the neuromodulatory actions of Ginkgo biloba leaf extract were pinpointed to many proteins that were significantly overexpressed in the hippocampus and cortex[29]. It indicated that Ginkgo biloba leaf extract prevents cognitive impairment of neurodegenerative disease by different mechanism.
